# Effects of Lasers and Their Delivery Characteristics on Machined and Micro-Roughened Titanium Dental Implant Surfaces

**DOI:** 10.3390/bioengineering7030093

**Published:** 2020-08-11

**Authors:** Thomas Fenelon, Mahmoud M. Bakr, Laurence J. Walsh, Roy George

**Affiliations:** 1 School of Dentistry and Oral Health, Queensland, Griffith University, Queensland 4215, Australia; tafenelon@gmail.com (T.F.); m.bakr@griffith.edu.au (M.M.B.); 2 School of Dentistry, The University of Queensland, Queensland 4006, Australia; l.walsh@uq.edu.au

**Keywords:** ablation, dental implants, Er:YAG, Nd:YAG, surface roughness, titanium

## Abstract

The aim of the study was to investigate the effects of neodymium: yttrium aluminium garnet (Nd:YAG) (1064 nm) and erbium: yttrium aluminium garnet (Er:YAG) (2940 nm) laser energy on titanium when delivered with conventional optics (focusing handpieces or plain ended optical fibres) or with a conical tip. Machined and micro-roughened implant discs were subjected to laser irradiation under a variety of energy settings either dry (without water) or wet (with water). Samples were scanned using a 3D non-contact laser profilometer and analysed for surface roughness, volume of peaks and the maximum diameter of the ablated area. Conical tip designs when used with both lasers showed no surface effect at any power setting on both machined and micro-roughened implant surfaces, regardless of the irrigation condition. When used with conventional delivery systems, laser effects on titanium were dose related, and were more profound with the Nd:YAG than with the Er:YAG laser. High laser pulse energies caused surface fusion which reduced the roughness of micro-roughened titanium surfaces. Likewise, repeated pulses and higher power densities also caused greater surface modifications. The presence of water reduced the influence of laser irradiation on titanium. It may be concluded that conical fibres can reduce unwanted surface modification, and this may be relevant to clinical protocols for debridement or disinfection of titanium dental implants.

## 1. Introduction

Titanium and its alloys are used commonly for dental implants. After insertion of a titanium dental implant, the accumulation of a dental plaque biofilm and an adverse host inflammatory response to this biofilm can lead to peri-implantitis, a condition where the bone around the implant is resorbed, and where if untreated the implant may be lost. Conventional treatments for peri-implantitis have included [1] mechanical debridement of the titanium implant surface using hand, sonic and ultrasonic scalers with plastic, metal or carbon fibre tips, air–powder devices, or pulsed infrared lasers; [2] disinfection using antiseptics, photothermal laser disinfection or photodynamic therapy, [3] local or systemic antibiotics, and [4] combinations of the above methods [3,5,6]. While the current literature describes many possible treatment modalities for peri-implantitis, there is a lack of high-quality evidence to suggest that any one approach could be considered a gold standard [7].

Lasers have been increasingly utilised in dentistry for the treatment both of periodontitis and peri-implantitis [8,9,10]. The neodymium: yttrium aluminium garnet (Nd:YAG) [11] the erbium: yttrium aluminium garnet (Er:YAG) [12,13], the erbium, chromium: yttrium, scandium, gallium, garnet (Er,Cr:YSGG) [14,15], the carbon dioxide (CO_2_) laser [11,16] and various diode lasers [17,18] have been used for periodontal applications. The Er:YAG laser has shown the most promising results in non-surgical periodontal therapy, with outcomes for laser debridement of the roots of teeth similar to those seen with mechanical debridement [13,19].

In the treatment of peri-implantitis, the erbium family of middle infrared lasers (Er:YAG and Er,Cr:YSGG) has received the most attention. The laser energy from both is absorbed strongly by water [20]. The Er:YAG laser has strong bactericidal effects against periodontal pathogens, and also inactivates bacterial toxins such as lipopolysaccharide [13,21]. When used on the roots of teeth, the Er:YAG laser can effectively remove plaque and calculus from implant surfaces without causing excessive heating of the adjacent bone [22,23].

Several studies have reported no significant change in the surface roughness of implants irradiated with Er:YAG lasers at intensities from 60 mJ/pulse to 180 mJ/pulse (average powers less than 1 Watt) and application times of up to 2 min [24,25,26]. Park et al. (2012) found, however, that alterations to titanium implant surfaces occurred if they were irradiated at average power settings over 2W for 30 s [27]. The nature of the laser interaction with the titanium surface of a dental implant may vary according to the surface topography. Titanium surfaces may be machined, sandblasted and acid-etched, titanium plasma sprayed, or anodic-oxidised. The surface treatment influences how the implant surface interacts with the adjacent bone [24,28]. Surface alterations such as melting of surface projections may increase with increasing laser energy and exposure time [22,26]. For example, surface alterations to machined and anodised titanium implants have been shown with Er,Cr:YSGG irradiation for 30 s at average power settings over 3W [27]. Accordingly, there is an interest in methods to reduce any adverse effect of infrared laser energy on titanium surfaces, either by altering the laser delivery system or by using water irrigation during laser irradiation.

The effect of any given laser depends not only on the absorption of the laser energy into the substrate but also on delivery system used, which changes the power density and may in some cases also change the pulse shape [22]. With middle infrared lasers, the delivery system could be an articulated arm, a hollow waveguide or a rare-earth element (germanium or gallium) containing fibre [29]. This would then be attached to a fibre tip, a handpiece with a focusing lens and a window or a hollow tip, depending on the system being used. When optical fibres are used for the distal part of the delivery system, factors such as the design of the fibre tip (e.g., plain, conical, or side firing) also influence the spatial distribution of laser energy onto the target [29].

Other factors that may influence the effect of laser energy on a substrate include the water-layer thickness if a cooling water mist spray is used [30], the pulse energy and pulse shape [31], the spot size [32] and the pulse duration [33]. Variations in the target may also influence the laser–target interaction, by changing the extent of reflection versus absorption [34]. This point is particularly relevant to titanium dental implants given the wide variety of surfaces available [24,27]. Any safe clinical protocol for dental use must consider how to minimize thermal stress on the alveolar bone and periodontal soft tissues [35]. A water coolant will reduce the temperature rise on the substrate. However, an excessive water film thickness may decrease absorption of laser energy by the substrate [36]. Suggested optimal water flow rates range from 1 to 2 mL/min [36] to 6.75 mL/min for ablation of dental enamel [37].

The primary aim of this study was to assess the effect of conical fibre tips versus conventional laser delivery systems (plain fibres and focusing handpieces) on the nature of the interaction of infrared laser energy with titanium. A secondary aim was to assess the influence of water irrigation on the interaction of laser energy with titanium. The primary laser of interest was the Er:YAG laser (2940 nm). An Nd:YAG laser (1064 nm) was included in the study as a positive control, since it is known to interact strongly with titanium and many other metals. It was hypothesised that the extent of surface modification of titanium surfaces would be reduced by the use of conical fibre tips and by the presence of water.

## 2. Materials and Methods

### 2.1. Lasers and Optical Fibres

Two free running pulsed laser systems were used: Nd:YAG (dLase 300, American Dental Laser, Fremont, CA, USA) and Er:YAG (Key 3, KaVo, Biberach, Germany). The Nd:YAG laser was fitted with a 200 μm plain glass fibre (BioLitec, Winzelaer, Germany) which was used either with a plain (cleaved) end, or after undergoing a conical honeycomb modification through a process of etching and abrasion as described previously [38] to increase lateral emission from the tip. With the Er:YAG laser, a germanium oxide-based fibre was used, which was then attached to either a focusing handpiece (model 2060) with a sapphire window, or a plain fibre handpiece (2062) with a 200 μm fibre that was used either with a plain (cleaved) end, or after undergoing the same conical honeycomb modification.

### 2.2. Titanium Discs

Discs of grade IV commercially pure titanium (Ti), as used in dental implants, were used (Dentsply Friadent GmbH, Steinzeugstr, Mannheim, Germany). These discs were 10 mm in diameter. The discs had a plain machined surface or had been micro-roughened by the manufacturer using grit-blasting and thermal etching to impart the desired microstructure for dental implants (Ankylos^®^ with plus surface, Dentsply Friadent GmbH, Steinzeugstr, Mannheim, Germany). Discs were cleaned with ethanol prior to the study to remove traces of machining oil or debris.

### 2.3. Experimental Setup

Titanium discs were subjected to laser irradiation under a variety of experimental conditions. A standardized setup was used, with the distal part of the laser delivery system mounted perpendicular to the surface of the titanium disc with a laboratory jig at a constant distance of 1.0 mm from the terminus of the delivery system to the target for the 200 μm fibre tips, and 10 mm (the focal point) for the Er:YAG laser-focusing handpiece. Multiple laser treatments were undertaken on non-exposed regions of each disc.

To explore the effect of a surface film of water, discs were irradiated dry (without water) or wet (with water). For the latter, a micro-pipette was used to transfer a 10 μL droplet of de-ionised water onto the surface of the discs immediately before laser exposure. 

### 2.4. Experimental Groups

The study followed a matrix design, and involved two different laser types (Er:YAG or Nd:YAG), two different fibre tip designs (plain or conical), and two different irrigation conditions (wet or dry). With the Er:YAG laser, a focusing handpiece was available, so this was also included as a further experimental group. Untreated discs and untreated areas of discs served as controls. The experimental groups are summarized in Table 1. The intention was to keep laser parameters within the range that would be used in clinical practice for implant debridement or disinfection, hence laser powers were kept at or below an average power of 3.0 watts (e.g., with the Nd:YAG laser, 100 mJ/pulse at 20 Hz gives an average power of 2.0 W, while 150 mJ/pulse at 20 Hz gives 3.0 W).

### 2.5. Emission Measurements

To inform the analysis, the actual emitted laser energy was measured for each laser and with each relevant delivery system. These measurements were taken at a fixed distance of 10 mm from the distal end of the delivery system, using a power meter (Nova II, Ophir Optronics, North Andover, MA, USA). The power density (W/cm^2^) was recorded over three seconds and the average value was used (Table 2).

### 2.6. Surface Profilometry

Surface analysis was conducted using a non-contact 3D laser profilometer (Talysurf CCI MP, Taylor Hobson, Leicester, England) using a 20× lens. Profilometric analysis was completed only for samples where laser irradiation produced a visible zone of ablation. Surface roughness was measured for all samples. This parameter quantifies the Z-axis perpendicular to the analysed surface (according to ISO 25178) [39]. The profilometer was calibrated at the beginning of each day of measurement using the manufacturer’s standard reference. The profilometric images produced were analysed using TalyMap software, Platinum version (Taylor Hobson, Leicester, England). To ensure a uniform method of data collection and analysis, a template was created within the TalyMap software, and applied to each sample, so that surface characteristics could be compared before and after laser irradiation of the same sample. Profilometric images were processed to determine the mean surface roughness (Sa), the volume of peaks within the ablated area, and the maximum diameter of the ablated area.

### 2.7. Analysis of Results

The surface roughness (Sa) of each sample before and following irradiation was compared using paired sample t-tests, for each of the gradations in laser parameters. Effects of irrigation on surface roughness, ablation zone volume and maximum diameter were compared for each laser parameter using a similar approach. Within each treatment group, the effects of laser parameters surface roughness, crater volume and maximum crater diameter were assessed for normality using the Kolmogorov–Smirnoff test and then analyzed by using ANOVA, with Bonferroni post-hoc tests. The level of significant was set at α = 0.05.

## 3. Results

### 3.1. Surface Roughness

#### 3.1.1. Control Group 

Prior to any laser treatments, the surface roughness of machined (smooth) surfaces (M = 0.23 µm, SD = 0.03) was significantly less than that of roughened surfaces (M = 2.73 µm, SD = 0.45), as supplied by the manufacturer (*p* = 0.0002).

#### 3.1.2. Group 1—Er:YAG Laser with Focusing Handpiece 

**Figure 1:**
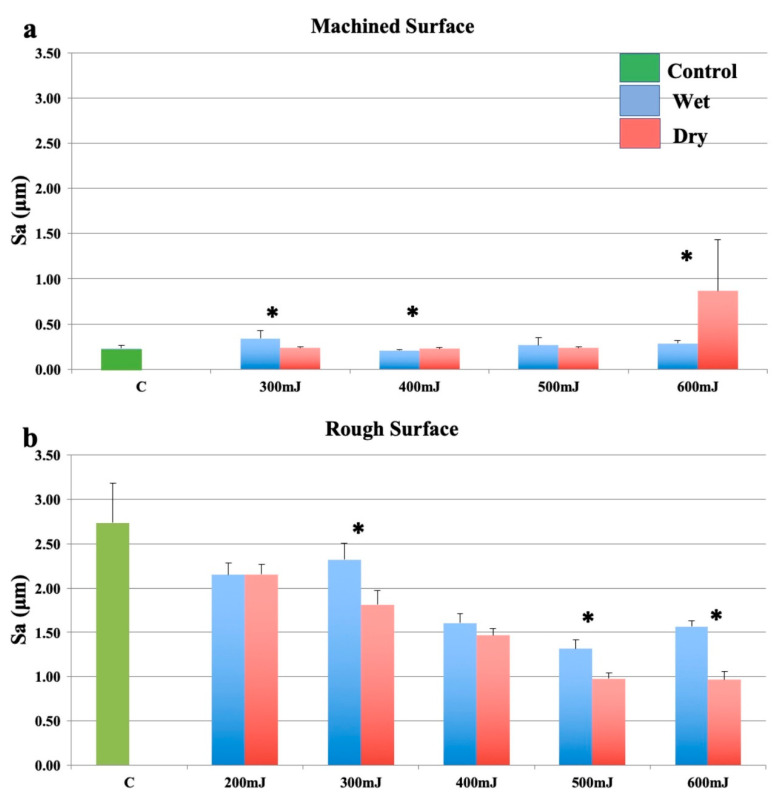
Effects of the Er:YAG laser with a focusing handpiece (2060). (**a**): Machined surfaces. (**b**): Roughened surfaces. Bars show controls (green), wet surfaces (blue), and dry surfaces (red). * indicates a significant difference between wet and dry conditions (*p* < 0.05).

Changes in mean surface roughness for machined (smooth surface) and rough surface samples irradiated with the Er:YAG laser using a focusing handpiece are shown in Figure 1. For smooth surfaces, there was significant alteration to surface roughness. With significantly greater roughness seen in the wet group when compared to the dry group at lower energy settings. This observation change between dry and wet ablation changed with increase energy settings (Figure 1a). Rough surfaces became progressively smoother as laser pulse energy increased, under both wet and dry conditions (Figure 1b). For the rough surfaces, with most laser parameters that were used, the surface smoothening was significantly greater when the sample was dry than when it was covered with a water film.

#### 3.1.3. Group 2—Er:YAG Laser with an Optical Fibre 

Conical fibre tip designs when used with the Er:YAG laser showed no surface effect at any laser parameter setting on both machined and micro-roughened implant surfaces, regardless of the irrigation condition. These groups are therefore not discussed further.

**Figure 2:**
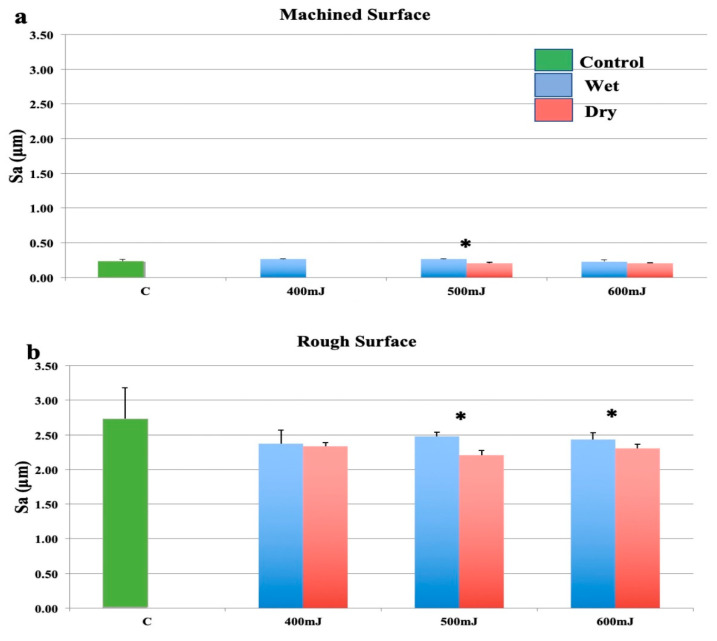
Effects of the erbium: yttrium aluminium garnet (Er:YAG) laser with a plain fibre tip held in a 2062 handpiece. (**a**): Machined surfaces. (**b**): Roughened surfaces. Bars show controls (green), wet surfaces (blue), and dry surfaces (red). * indicates a significant difference between wet and dry conditions (*p* < 0.05).

As shown in Figure 2a, when using an optical fibre with a plain (cleaved) end, there was no significant change to smooth surfaces at any energy setting regardless of irrigation condition compared to the control group. The surface roughness at 400 mJ was not assessed for the dry samples as no surface modification could be recorded. These samples should therefore be considered similar to controls. Wet ablation significantly increased the surface roughness at 500 mJ and displayed a trend to increased roughness at 600 mJ, although this was not statistically significant.

The reduction in surface roughness for rough samples (Figure 2b) was less marked than when the focusing handpiece (2060 handpiece) was used at the same settings. There was a slight decrease in surface roughness at all power settings, however, this was not statistically significant. Comparing the effect of water, wet surfaces were significantly rougher when ablated at 500 mJ/pulse and 600 mJ/pulse (Figure 2b). The presence of water generally reduced the laser induced surface modification. 

#### 3.1.4. Group 3—Nd:YAG Laser with an Optical Fibre

Conical tip designs, when used with the Nd:YAG laser showed no surface effect at any laser parameter setting on either machined or micro-roughened implant surfaces, regardless of the irrigation condition. These groups are therefore not discussed further.

**Figure 3:**
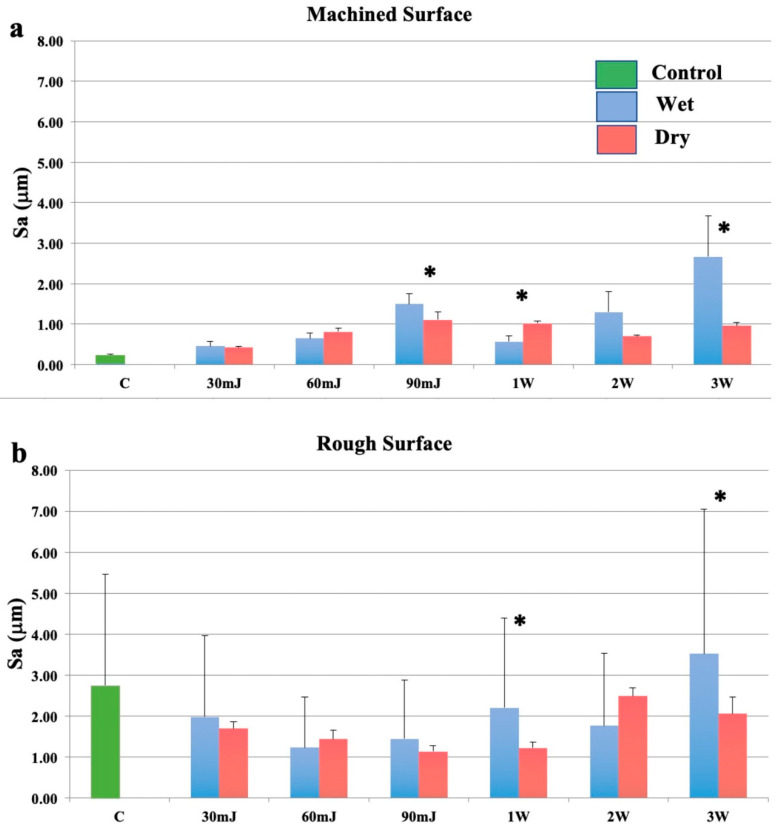
Effects of the neodymium: yttrium aluminium garnet (Nd:YAG) laser with a plain fibre tip. (**a**): Machined surfaces. (**b**): Roughened surfaces. Bars show controls (green), wet surfaces (blue), and dry surfaces (red). * indicates a significant difference between wet and dry conditions (*p* < 0.05).

Exposure to the Nd:YAG laser increased the surface roughness of smooth Ti discs under wet and dry conditions, and this effect was variably attenuated by a film of water (Figure 3a). On rough surfaces, a bimodal effect was seen, with a reduction in roughness at 30–90 mJ/pulse delivered as single pulses (Figure 3b). Similarly, surface roughness was decreased following exposure of rough surfaces to the Nd:YAG laser at all but the highest laser energy, however, this increase was not statistically significant. This effect was compounded by the fact that the highest pulse energy settings (100 and 150 mJ/pulse) were used at 20 Hz. 

### 3.2. Volume of Peaks in the Ablation Zone

#### 3.2.1. Group 1—Er:YAG Laser with Focusing Handpiece

The total volume of peaks within the ablation zone was measured for samples treated at 300 mJ and above, as these gave a measurable zone of ablation. For smooth discs, there was no significant relationship between pulse energy and the volume of peaks, and this outcome variable was not influenced by the presence of water. For rough surface discs, there was no significant relationship between pulse energy and the volume of peaks for wet conditions, but there was a significant positive relationship for dry conditions. Water reduced the peak volume only at 300 mJ. 

#### 3.2.2. Group 2—Er:YAG Laser with Optical Fibre

Measurements of volume or of the diameter of the ablation zone were not possible as no visible zone of ablation was produced on the titanium surface. 

#### 3.2.3. Group 3—Nd:YAG Laser with Optical Fibre

On both machined surfaces and roughened surfaces, there was a significant positive relationship between pulse energy and the volume of peaks for both wet and dry conditions. The presence of water had a variable influence on smooth samples, with a trend to decrease the volume of peaks for rough surfaces at higher pulse energy settings.

### 3.3. Maximum Diameter of the Zone of Ablation

#### 3.3.1. Group 1—Er:YAG Laser with Focusing Handpiece

For smooth surfaces, under wet conditions, power level did not influence the maximum diameter of the ablation zone. Under dry conditions the diameter was significantly decreased at 400–600 mJ. For rough samples, 500 mJ and 600 mJ power resulted in significantly greater diameter of the ablation zone. 

#### 3.3.2. Group 2—Er:YAG Laser with Optical Fibre

Measurements of diameter of the ablation zone for Group 2 was not possible as no visible zone of ablation was produced on the titanium surface.

#### 3.3.3. Group 3—Nd:YAG with Optical Fibre

The diameter of the ablation craters increased with pulse energy, on both smooth and rough surfaces. The presence of water reduced the diameter of the zone of ablation for both surface types. 

## 4. Discussion

This study provides several insights into the interactions between near infrared and middle infrared laser energy and titanium, with the former being strongly absorbed, and the latter only weakly absorbed. The titanium discs provided by the manufacturer are consistent with the surfaces provided on implants for clinical use, with the smooth surface being typical for abutment components, and the roughened surface being representative of the surface of the implant fixture below the alveolar bone crest. Indeed, the roughened discs displayed almost twelve times the roughness of machined discs. The purpose of this implant surface modification is to increase the surface area available for apposition of bone during osseointegration of the implant [40,41]. The titanium discs used in this study with a microscopically roughened surface had a greyish tint. The irregular nature of the microscopically roughened surface could be the reason why more variation was seen for laser effects on this surface type than for the machined surface, which was more regular in its topography.

The Nd:YAG laser was used as a positive control in this study. While this laser induced surface modifications to both types of titanium surface when the plain optical fibre tip was used, no modifications occurred when the modified conical honeycomb tip was used. Based on this, the original hypothesis that the use of the conical tip would reduce the adverse effects of laser energy on the titanium surface is supported.

Despite previous research indicating the deleterious effects of Nd:YAG lasers on implant surfaces [42,43,44], the current findings indicate that even a strongly absorbed laser wavelength may have minimal effects on the substrate when it is dispersed widely by the delivery system. Indeed, the Nd:YAG laser used with a plain optical fibre tip and a low pulse energy (below 30 mJ) may be appropriate for post debridement disinfection around titanium implants. Using a low pulse energy would reduce the risk of severe surface modifications, such as melting, fusion and cratering. As was expected, when used with plain fibre tips on rough implant surfaces, the Nd:YAG laser at low pulse energies generally reduced the roughness of the surface because of melting. With higher energy pulses, cratering occurred in a proportional fashion to the delivered energy. The influence of water on the interaction of Nd:YAG laser energy with the roughened titanium surface was variable, with no strong trends seen. The absorption of Nd:YAG laser radiation in water is very low, hence any changes seen could be due to altered reflection or scatter from the surface, as well as from cooling effects. The impact of irrigation condition requires further clarification with the use of continuous water spray.

With regard to the Er:YAG laser, its effects on titanium surfaces were less than those of the Nd:YAG laser. Overall, the current findings are consistent with previous research demonstrating no significant surface effects following irradiation with the Er:YAG laser at up to 140 mJ/pulse at 10 Hz on roughened (SLA surface) implants [24] and on machined and anodised surface implants [27] when examined with white light interferometry and scanning electron microscopy. Laser profilometry measurements of surface roughness revealed that the mean 3D surface roughness (Sa) of smooth titanium could be increased by exposure to the Er:YAG laser, but only when high pulse energies were used. Compared to previous research, the present study used relatively high pulse energy settings for the Er:YAG laser with optical fibre delivery. Despite this, minimal surface modification was caused on smooth or roughened surfaces. Based on the present findings, it can be concluded that provided a low pulse energy is used, no adverse effects on the smooth titanium implant surfaces should occur. The Er:YAG laser used in the present study had a pulse duration of 250–350 microseconds. If a much shorter pulse duration was used, the higher peak power that occurs with this could, however, pose a risk of causing surface alterations. The parameter of pulse duration requires further investigation.

With regard to the role of water, under clinical conditions, typical pulse energies are in the order of 50–200 mJ, and the Er:YAG laser is used with a water mist spray. The Er:YAG laser, when used at low pulse energy with a water mist spray, was found to effectively remove experimentally induced biofilms from SLA implant surfaces at 80–100 mJ/pulse (10 Hz), without significant alteration to the surface [45,46]. In the present study, as the water was not applied continuously, both heating and evaporation of the water may have occurred, thereby reducing the effectiveness of the water in reducing thermal changes on the titanium surface. 

The results of the present study also show the effect of laser spot size and thus power density on Er:YAG laser effects on titanium, with the focusing handpiece causing significant effects for the same pulse energy, compared to the plain optical fibre. Plain fibre ends give a beam dispersion of around 20°. Moreover, the gallium doped endodontic fibre used in the current investigation attenuates the laser energy within the fibre, to a greater extent than occurs with the sapphire window of the focusing handpiece [47]. Any attenuation of laser energy by absorption in the optical fibre, reduced transmission with repeated use, or degradation of the fibre tip will reduce the laser effect, and this will result in decreased surface temperatures and thus decreased propensity for damage to titanium surfaces [22]. For the roughened samples irradiated with the Er:YAG and focusing handpiece, a significant relationship between increasing pulse energy and decreasing surface roughness was seen, regardless of irrigation, demonstrating that significant modification of these surfaces had occurred. It can be concluded, therefore, that using a focusing handpiece would not be appropriate for clinical implant therapy. 

An important variable that needs to be taken into consideration is the spot profile. The present laser had a Gaussian beam profile. Some dental Er:YAG lasers have complex beam profiles because of multimode operation, and this spatial inhomogeneity may influence the geometry of surface craters on the substrate [31,48]. The water flow rate for irrigation varies between dental Er:YAG lasers. Higher water flow rates may cause greater attenuation of laser effects on titanium surfaces, because of higher water film thickness, as well as a greater cooling action, which may prevent melting of the titanium surface [22,36]. In the present study, the water film was stationary rather than moving, and was not replenished continuously. Despite this, surface modifications were generally reduced under wet conditions. It is reasonable to expect that surface temperature, and thus potential for implant surface modification, would be decreased with the use of continuous air–water spray from the laser handpiece as would be used routinely with an Er:YAG laser in clinical practice. 

A key point of interest in the current study was the lack of appreciable surface modification or deleterious effects from either laser when modified conical fibre tips were used. This can be explained by the reduced beam intensity in a frontal (distal) direction (by as much as 49%) and correspondingly greater lateral emission of laser energy [38,49,50]. This aspect may be relevant to the use of lasers for periodontal disinfection following the debridement of dental implants and root surfaces. Such conical laser tips may be advantageous in directing laser energy laterally onto the implant surface, as well as against the soft tissue walls of the periodontal pocket, and may be particularly useful in accessing narrow bony defects adjacent implant fixtures. The clinical efficacy and applicability of modified optical fibre tip designs need to be investigated further in clinical trials. 

The generalization of the current findings to the clinical setting should be performed with caution. The 90-degree angulation of the laser beam to the titanium surface used in the study would not necessarily be the same as for clinical practice. The results would also vary for other brands of lasers, because of differences in pulse shape and pulse duration. Hence, the current results may not be directly applied to other brands of Nd:YAG and Er:YAG laser systems on the market. Further, the impact of crevicular fluid, blood and saliva may not be the same as for water. The presence of plaque biofilm and calculus on laser interactions with titanium was outside the scope of this laboratory study.

## 5. Conclusions

Pulses of laser energy from Er:YAG and Nd:YAG lasers may modify the surface characteristics of machined and roughened dental implants, depending on the pulse energy and the power density. The Er:YAG laser delivered using a plain fibre tip was associated with minimal surface modification, while the Nd:YAG laser used at similar parameters caused considerable changes. In contrast, when a modified conical tip was used with either laser type, no surface melting or cratering occurred on either machined or micro-roughened titanium surfaces. Using such tips for implant debridement or disinfection may help prevent undesirable surface modification. 

## Figures and Tables

**Table 1:** Experimental design.

	Panel Setting	Implant Surface and Fibre Tip Design Combinations							
		Machined Surface				Micro-Roughened Surface			
		Plain		Conical		Plain		Conical	
Group 1 Er:YAG with fibre in 2062 handpiece	400 mJ/1Hz 500 mJ/1Hz 600 mJ/1Hz	Wet	Dry	Wet	Dry	Wet	Dry	Wet	Dry
Group 2 Er:YAG 2060 Focusing handpiece	200 mJ/1Hz 300 mJ/1Hz 400 mJ/1Hz 500 mJ/1Hz 600 mJ/1Hz			N/A				N/A	
Group 3 Nd:YAG with fibre	30mJ/10Hz 60 mJ/10Hz 90 mJ/10Hz 1W/20Hz 2W/20Hz 3W/20Hz			Wet	Dry			Wet	Dry
Group 4 Control	Non-irradiated	N/A		N/A		N/A		N/A	

**Table 2:** Power density for laser systems.

	Panel Setting	Power Density (W/cm^2^)	
		Conical Fibre Tips	Plain Fibre Tips
Er:YAG fibre in 2062 handpiece	400 mJ/1Hz 500 mJ/1Hz 600 mJ/1Hz	5.40 6.45 7.60	11.50 13.25 15.45
Er:YAG 2060 focusing handpiece	200 mJ/1Hz 300 mJ/1Hz 400 mJ/1Hz 500 mJ/1Hz 600 mJ/1Hz	N/A	6.75 8.55 11.35 14.65 18.45
Nd:YAG	30 mJ/10Hz 60 mJ/1Hz 90 mJ/1Hz 50 mJ/20Hz (1 W) 100 mJ/20Hz (2 W) 150 mJ/20Hz (3 W)	9.90 16.50 25.00 29.50 59.00 81.00	12.00 23.00 34.00 38.00 75.00 109.00
